# Modified Masquelet technique in children

**DOI:** 10.1016/j.cjtee.2021.09.002

**Published:** 2021-09-17

**Authors:** Ravi Mittal, Siddharth Jain

**Affiliations:** Department of Orthopaedics, All India Institute of Medical Sciences, New Delhi, India

**Keywords:** Masquelet technique, Induced membrane, Children, Gap non-union, Free fibula graft

## Abstract

Masquelet technique is one of the modalities for the treatment of long bone defect. Using cancellous bone graft to fill the bone defect is always a concern in children due to the small size of their iliac crest and open growth plate. We reported a case of 13-year-old male who presented with gap non-union of middle third of tibia. We applied a modified Masquelet technique by using only the cortical fibular graft instead of cancellous bone to fill the space surrounded by induced membrane. Fibula was used as a nonvascularized strut graft and matched stick graft to achieve complete union. We concluded that nonvascularized fibula grafting is an easy and effective option to fill the bone defect in children in the second stage of Masquelet technique.

## Introduction

Bone defect in a long bone can result from debridement of avascular and comminuted bone fragment in open fractures, sequestrectomy in chronic osteomyelitis or resection of tumour.^1^ There are various methods to treat this gap in a long bone. Autogenous cancellous bone grafting is a commonly used technique but has some limitations when the bone defect is large.^2^^,^^3^ This problem is more acute in children with open growth plate and small iliac crest. Free vascularized fibular grafting offers adequate reconstruction of large bone defect but it is a technically demanding procedure.^4^ Bone transport with Ilizarov fixator or limb reconstruction system has issues of complexity of applying implant, lengthy treatment and poor patient compliance.^5^ Masquelet technique or induced membrane technique is another popular treatment modality for bone defect.^6^^,^^7^ It is a two-stage technique where cancellous bone is used in the second stage to fill the bone gap surrounded by induced membranes.^8^^,^^9^ Children tolerate Masquelet technique better than Ilizarov bone transport technique or other external fixator used for bone transport.^9^^,^^10^ Free fibular strut graft is another good option as it provides not only adequate size graft but also high potential of donor site regeneration.^11^ We reported a case where we used nonvascularized free fibula graft instead of autogenous cancellous bone graft in the second stage of Masquelet technique.

## Case report

**Fig. 1:**
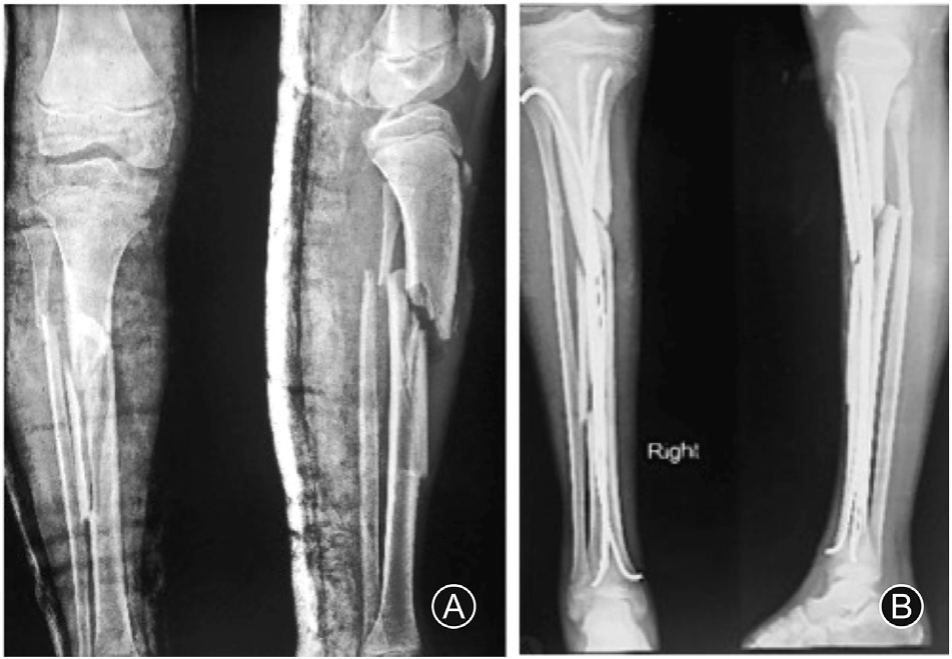
(A) Fracture of middle third of tibia and fibula; (B) Fracture treated with elastic nails in tibia.

**Fig. 2:**
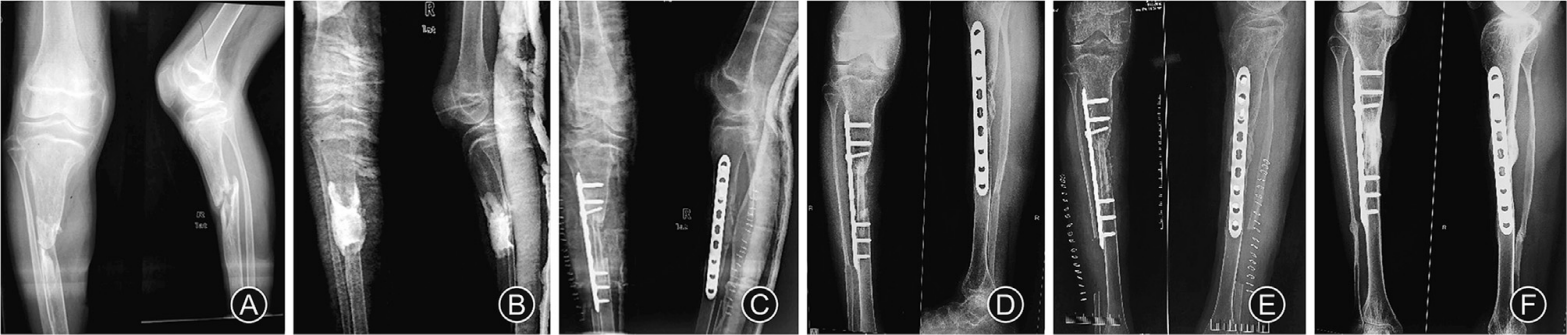
(A) Gap non-union of tibia after debridement and removal of nails; (B) Antibiotic cement spacer in place after debridement in the first stage of Masquelet procedure; (C) Cement spacer was removed and plating of tibia done in the second stage of Masquelet procedure performed after 6 weeks of the first stage. Ipsilateral fibula used as intra-medullary graft and match stick graft in the gap; (D) Union of the graft occurred after 12 months but it was not thick enough to allow weight bearing. Regeneration of ipsilateral fibula occurred; (E)Ipsilateral fibula harvested for second time and used as match stick graft at the end of 12 months; (F) Complete union with adequate thickness of the healed graft after 18 months. Fibula has regrown after the second harvest.

**Fig. 3:**
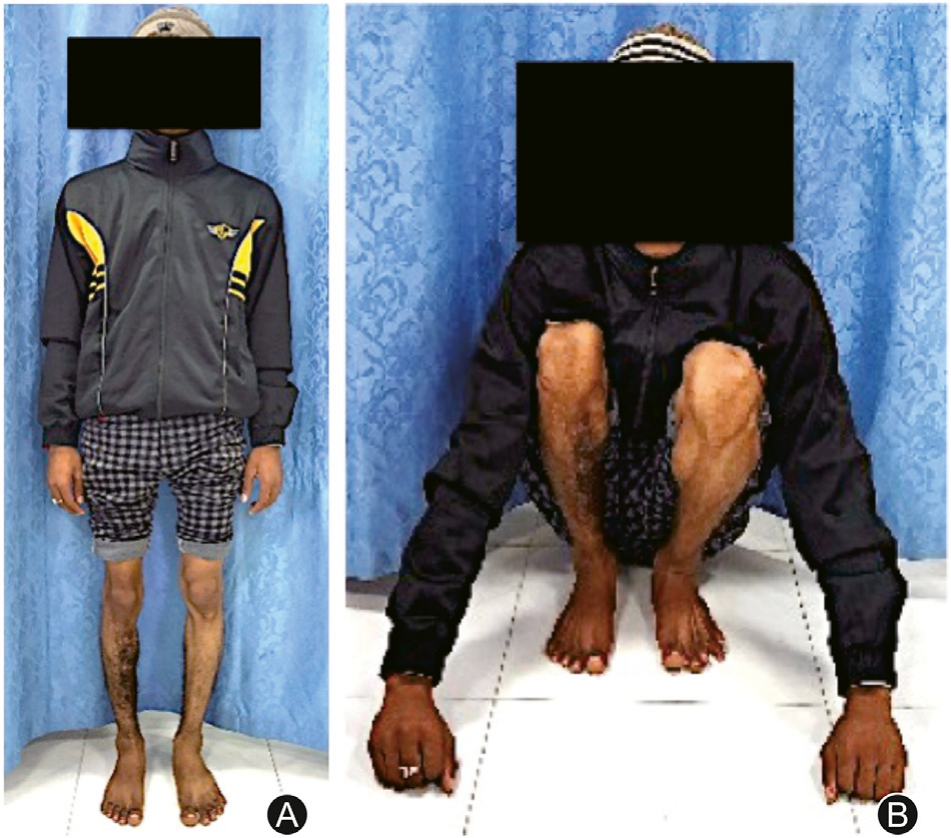
Full extension and flexion of the knee with good ankle movements at the end of 24 months.

A 13 years old male sustained a fracture of the right sided tibia and fibula for which elastic nailing of tibia was employed (Fig. 1). The fracture was infected, which was managed by implant removal and debridement. The patient presented to us with gap non-union of tibia in the middle third without any active infection (Fig. 2A). After thorough clinical and radiological examination, Masquelet procedure was planned for him. In the first stage, through an anterolateral approach to tibia, freshening of the bone ends was done to bleeding bone. Tissue samples were sent for culture. The bone defect which was now approximately 5 cm in length was filled with gentamycin bone cement spacer. Immobilization was done with thick above knee cast (Fig. 2B). The culture was positive for *Staphylococcus aureus* which was sensitive to Ciprofloxacin. The patient was given Ciprofloxacin for 3 weeks. Six weeks later the second stage of the surgery was planned. On exposure we found a white coloured membrane around the cement spacer. The cement spacer was removed while preserving the membrane. The ipsilateral middle third of fibula of length 9 cm was harvested through postero-lateral approach. It was ensured that proximal and distal tibiofibular joint remained unaffected. The fibula graft was cut into 2 pieces with length of 7 cm and 2 cm. The longer segment of the fibula graft was used as intramedullary graft in the tibial defect and internal fixation was done using 4.5 mm locking compression plate. Before placing the graft, both ends of the non-union side were adequately freshened. The shorter segment of fibula graft was cut into thin matchstick grafts and placed around intramedullary graft. No cancellous graft was used (Fig. 2C). The white coloured membrane was closed meticulously. Ciprofloxacin was given for 2 weeks. Patient was kept non-weight bearing and range of motion exercises and isometric quadriceps exercises were started on third postoperative day. Patient was followed up every 2 months till 1 year. Graft healing was seen at 12-month but graft segment was not of adequate thickness (Fig. 2D). So bone grafting was again advised to the patient. As the ipsilateral fibula had regrown, we were able to harvest 5 cm of ipsilateral fibula again. It was cut in to small pieces and placed around the previous graft (Fig. 2E). Complete union with adequate thickness was observed after 6 months. Fibula was found to be grown again (Fig. 2F). Patient was followed up for 2 years and he showed full range of motion at knee and ankle on the same side with normal gait (Fig. 3).

## Discussion

There is a lack of consensus to guide the treatment for large bone defect in long bones. Free nonvascularized fibula grafting is easier and requires less surgical time compared to free vascualrized fibula grafting.^12^^,^^13^ But stress fracture, non-union and donor site morbidity remain as problems with this technique.^14^^,^^15^ The union can be enhanced by stable fixation and placing the fibula graft in a biologically active environment that promotes bone growth and prevents bone resorption. Induced membranes in Masquelet technique can provide such an environment. Stress fractures of fibula strut graft can occur when it is excessively loaded before it hypertophies.^16^ This can be prevented by avoiding weight bearing and placing implants that off-load the graft. Donor site morbidity would be minimal if the harvested segment of fibula re-grows. And various authors have reported that fibular regeneration occurs in more than 75% cases at follow-up.^11^^,^^17^

Masquelet technique is a two-stage method that uses induced membranes with filling of the cancellous bone graft in second stage to manage large bone defects.^6^ In the first stage after debridement and placement of antibiotic-impregnated cement spacer, an interval of 6–8 weeks is needed for the formation of a biologically active membrane. This membrane has properties of promoting bone growth and preventing graft from resorption.^7^ In the second stage, the cement spacer is removed and autogenous cancellous bone is placed. Although its exact aetiology is not known, bone graft resorption has been seen in children. The probable causes include inadequate fixation, tumour or infection recurrence and delay of the second stage for more than 8 weeks.^10^ If the percentage of the bone substitute or allograft exceeds 40% of the autograft, the risk of non-union and graft resorption rises exponentially.^10^ This technique has made it possible to reconstruct bone loss up to 30%–50% of total bone length. Children tolerate Masquelet technique better than external fixator used for bone transport.^9^^,^^10^ Moreover, the time to healing is independent of the defect length in the Masquelet technique.^10^ Deep surgical-site infections and pin-tract infections are less common in Masquelet technique as compared to bone transport technique.^18^

The average time to bone union after the second stage was 9.5 months (range 5–25 months) when combined for all the pathologies. It was 7.8 months for congenital pseudoarthrosis, 8.0 months for benign tumors, 8.3 months for malignant tumors and 15.3 months for traumatic injuries.^9^^,^^18^^,^^19^ We achieved union in 12.0 months, when only intra-medullary fibula strut graft and small pieces of fibula were used without any cancellous bone. Moreover, the fibula regrew not once, but twice at the donor site.

We could not find any similar report in the literature where only cortical bone was used in the second stage of Masquelet technique without any cancellous bone. Good result was achieved by the combined advantages of Masquelet technique and nonvascularized free fibula grafting where the paucity of cancellous graft for the bone defect was managed by using fibula graft and the healing of fibula graft was enhanced by the use of induced membranes. We conclude that nonvascularized fibula grafting is an easy and effective option to fill the bone defect in children in the second stage of Masquelet technique.

## Funding

Nil.

## Ethical statement

This case report was exempted from ethical approval by Institute Ethics Committee, All India Institute of Medical Science.

## Declaration of competing interest

The authors declare that there is no conflict of interest.

## Author contributions

Ravi Mittal - performed the surgery and reviewed the manuscript. Siddharth Jain - worte the manuscript.
